# Correlated evolution between targets of pre‐ and postcopulatory sexual selection across squamate reptiles

**DOI:** 10.1002/ece3.2344

**Published:** 2016-08-18

**Authors:** Ariel F. Kahrl, Christian L. Cox, Robert M. Cox

**Affiliations:** ^1^ Department of BiologyUniversity of VirginiaCharlottesvilleVirginia22904; ^2^ Department of BiologyGeorgia Southern UniversityStatesboroGeorgia30460

**Keywords:** Phylogenetic comparative method, sexual size dimorphism, sperm competition, testis size

## Abstract

Sexual selection reflects the joint contributions of precopulatory selection, which arises from variance in mating success, and postcopulatory selection, which arises from variance in fertilization success. The relative importance of each episode of selection is variable among species, and comparative evidence suggests that traits targeted by precopulatory selection often covary in expression with those targeted by postcopulatory selection when assessed across species, although the strength and direction of this association varies considerably among taxa. We tested for correlated evolution between targets of pre‐ and postcopulatory selection using data on sexual size dimorphism (SSD) and testis size from 151 species of squamate reptiles (120 lizards, 31 snakes). In squamates, male–male competition for mating opportunities often favors large body size, such that the degree of male‐biased SSD is associated with the intensity of precopulatory selection. Likewise, competition for fertilization often favors increased sperm production, such that testis size (relative to body size) is associated with the intensity of postcopulatory selection. Using both conventional and phylogenetically based analyses, we show that testis size consistently decreases as the degree of male‐biased SSD increases across lizards and snakes. This evolutionary pattern suggests that strong precopulatory selection may often constrain the opportunity for postcopulatory selection and that the relative importance of each selective episode may determine the optimal resolution of energy allocation trade‐offs between traits subject to each form of sexual selection.

## Introduction

Sexual selection can be divided into two episodes that jointly contribute to the net opportunity for selection: precopulatory selection, which arises from variance in mating success, and postcopulatory selection, which arises from variance in fertilization success. When reproductive success is strongly influenced by the acquisition and monopolization of mates, precopulatory selection is the primary determinant of the net opportunity for sexual selection (Pischedda and Rice 2012; Rose et al. 2013; Pélissié et al. 2014). Conversely, when polyandry is high, the opportunity for postcopulatory selection can equal or exceed that for precopulatory selection (Collet et al. 2012; Devigili et al. 2015; Turnell and Shaw 2015). Because these two episodes of sexual selection occur sequentially, the outcome of precopulatory selection has the potential to shape the opportunity for postcopulatory selection (Preston et al. 2003; Hunt et al. 2009; South and Lewis 2012). For example, strong precopulatory selection that results in monopolization of females can reduce the opportunity for sperm competition (Preston et al. 2003; Fitzpatrick et al. 2012; Parker et al. 2013). Consequently, in taxa where precopulatory selection is associated with male–male contest competition and monopolization of females, species are predicted to fall along a continuum ranging from those that invest primarily in traits under precopulatory selection to those that invest primarily in traits under postcopulatory selection (Parker et al. 2013; Lüpold et al. 2014).

Consistent with this prediction, interspecific comparisons across several taxa have revealed that traits subject to precopulatory selection, such as weaponry and body size, often correlate negatively with traits subject to postcopulatory selection, such as testis size (Heske and Ostfeld 1990; Poulin and Morand 2000; Fitzpatrick et al. 2012; Dines et al. 2015; Dunn et al. 2015). In addition to the potential role of precopulatory selection in limiting the opportunity for postcopulatory selection, such negative interspecific correlations could also arise from (or be strengthened by) energetic trade‐offs between sexually selected traits (Moczek and Nijhout 2004; Simmons and Emlen 2006; Kelly 2008; Parker and Pizzari 2010; Yamane et al. 2010; Somjee et al. 2015). However, analyses in other taxa have found no evidence for negative interspecific correlations between targets of pre‐ and postcopulatory selection (Ferrandiz‐Rovira et al. 2014; Lüpold et al. 2014), and several studies have documented positive correlations (Wedell 1993; Lüpold et al. 2014). The reasons for such discrepancies are still unclear, emphasizing the need for further comparative analyses of this pattern (Lüpold et al. 2014).

Squamate reptiles (lizards and snakes) provide an intriguing group in which to test for correlated evolution between targets of pre‐ and postcopulatory selection because they often experience strong precopulatory selection on body size and other traits involved in territory defense and mate acquisition (Cox and Kahrl 2014), and because precopulatory selection for large male size is known to influence the direction and magnitude of sexual size dimorphism in this group (Cox et al. 2003, 2007). Postcopulatory selection is also known to favor increased testis size, relative to body size, in squamates (Todd 2008; Uller et al. 2010), as it does in many other taxa (Harcourt et al. 1981; Harvey and Harcourt 1984; Møller 1989, 1991; Heske and Ostfeld 1990; Gage 1994; Møller and Briskie 1995; Stockley et al. 1997; Hosken and Ward 2001; Rowe and Pruett‐Jones 2011). Multiple paternity appears to be the rule rather than the exception in squamates, occurring in over 50% of all clutches and in all 23 species examined by Uller and Olsson (2008). Hence, the extent to which males actually monopolize females via mate defense and territoriality is generally uncertain across this lineage. Moreover, some territorial squamates exhibit male‐biased SSD in association with high levels of multiple paternity (e.g., *Anolis sagrei*, Calsbeek et al. 2007), whereas other territorial species exhibit female‐biased SSD in association with low levels of multiple paternity (e.g., *Sceloporus undulatus*, Haenel et al. 2003). These observations suggest that simple classifications of mating system on the basis of territoriality may be of limited utility for assessing the interplay between pre‐ and postcopulatory sexual selection and also raise the question of whether squamates exhibit the same negative association between targets of pre‐ and postcopulatory selection that characterizes other lineages.

To answer this question, we tested for an interspecific correlation between the phenotypic targets of pre‐ and postcopulatory selection using a dataset of 151 species of squamate reptiles – the largest comparative dataset to explore this pattern in any lineage. We combined this dataset with a recent phylogeny of Squamata (Pyron et al. 2013) to test for correlated evolution between sexual size dimorphism (SSD), a consequence of precopulatory selection for large male body size, and testis size, a target of postcopulatory selection for sperm production. We predicted that SSD and testis size would be negatively correlated across species for either of two complementary reasons: (1) because strong precopulatory selection may limit the opportunity for postcopulatory selection; and (2) because energetic trade‐offs may limit the extent to which both traits can be simultaneously maximized.

## Methods

### Comparative dataset

We used estimates of SSD compiled by Cox et al. (2003, 2007) as the basis for our dataset and added measures of testis size from literature searches and unpublished collections (Appendix S1). We recorded mean body size (snout‐vent length, SVL) and mean testis size from additional published studies that we obtained by reviewing the literature on testis size and reproductive cycles. We only included species for which we could obtain estimates of testis size during the reproductive season and measures of SVL for adults of each sex. We supplemented this literature review with our own measures of SVL and testis size from 50 wild populations (Appendix S2). In total, our dataset included 151 species (120 lizards, 31 snakes) representing 16 squamate families (Appendix S1). For each species, we calculated an index of SSD as:

$$\text{SSD}=\frac{\text{SVL}\;\text{of}\;\text{larger}\;\text{sex}}{\text{SVL}\;\text{of}\;\text{smaller}\;\text{sex}},$$

expressed as a positive value when males are the larger sex and a negative value when females are the larger sex. For analysis, we log_10_‐transformed this index prior to assigning it a positive or negative value. Testis size was typically reported as either volume (mm^3^) or mass (mg), and we converted mass to volume using the density (1 mg/mm^3^) given by Licht and Pearson (1969). If only testicular dimensions were reported, we calculated volume using the formula:

$$\text{Volume}=\frac{4}{3}\pi a^{2}b,$$

where *a* is the radius of the width of the testis and *b* is the radius of its height. When seasonal patterns of testis size were reported, we used maximum testis size during the breeding season.

### Nonphylogenetic analyses

To test the prediction of a negative correlation between traits associated with pre‐ and postcopulatory selection, we used multiple regression with log_10_ testis size as the dependent variable and log_10_ SSD and log_10_ male SVL (to account for scaling) as independent variables. Because snakes and lizards differ dramatically in body shape and, consequently, in scaling relationships with SVL, we conducted these analyses separately within each group (Losos 1994), despite the fact that snakes comprise a derived clade nested within lizards (Pyron et al. 2013).

### Phylogenetic analyses

We used the phylogeny of Pyron et al. (2013), which provides branch lengths and a resolved topology for all species in our dataset, to test for correlated evolutionary changes in SSD and testis size in the R environment (R Development Core Team 2013). First, we removed all species that were not found in our dataset from the phylogeny using APE (Paradis et al. 2004). We used this pruned phylogeny to test for phylogenetic signal in both SSD and testis size using Blomberg's *K* (Blomberg et al. 2003) and Pagel's *λ*, implemented in Picante (Kembel et al. 2010) and phytools (Revell 2012), respectively. Blomberg's *K* is calculated using the variance/covariance matrix of the phylogenetic relationships among species. It is the ratio of the mean squared error of observed trait values versus the mean squared error of predicted trait values modeled under Brownian motion, with significance (*K *>* *0) calculated using simulation tests (Blomberg et al. 2003). If *K *<* *1, phenotypic variance is greater within clades than expected under Brownian motion, whereas if *K *>* *1, phenotypic variance tends to be relatively greater among clades (Blomberg et al. 2003). Pagel's *λ* defines the amount of phylogenetic signal, where *λ *= 0 corresponds to complete phylogenetic independence and *λ *= 1 indicates that traits vary across the phylogeny as predicted by Brownian motion (Pagel 1999). We used log‐likelihood ratio tests to determine whether values of Pagel's *λ* were significantly different from 0 and 1.

We tested for evolutionary associations between SSD and testis size using phylogenetic generalized least squares (PGLS) regressions. As described above, we conducted separate analyses for lizards versus snakes and also tested for relationships between SSD and testis size within any lineage of lizards represented by ≥8 species. In all analyses, we used a Brownian motion model of character evolution (Table 1, Table S4). As a complementary method, we also used an Ornstein–Uhlenbeck model of character evolution, but only had the statistical power to fit the model to lizards due to our limited sampling of snakes and other lineages (Table S3). We conducted these analyses in the R package APE (Harmon et al. 2008) with log_10_ testis size as the dependent variable and log_10_ SSD and log_10_ male SVL (to account for scaling) as independent variables. As a complementary approach, we also tested for a correlation between SSD and testis size using phylogenetically independent contrasts (PIC). In order to account for a size covariate using PIC, we followed the approach of Garland et al. (1992). First, we calculated contrasts of log_10_ SSD, log_10_ testis size, and log_10_ SVL using the pic function in APE (Harmon et al. 2008). Next, we positivized all contrasts with respect to SVL (Garland et al. 1992) and conducted an ordinary least‐square regression (forced through the origin) of the contrasts of log_10_ SSD regressed on the contrasts of log_10_ SVL, and of the contrasts of log_10_ testis size regressed on the contrasts of log_10_ SVL. Finally, we used an ordinary least‐square regression (forced through the origin) to test for a negative relationship between the residuals from each of these regressions, which represent phylogenetically independent and size‐corrected measures of SSD and testis size (Garland et al. 1992). We conducted these analyses separately for lizards and snakes.

**Table 1:** Summary of results from phylogenetic generalized least squares (PGLS) regression of log_10_ testis size on log_10_ sexual size dimorphism (SSD) with log_10_ mean male body size (snout‐vent length, SVL) as a covariate, using a Brownian motion model of character evolution. Analyses were conducted separately for lizards (*N* = 120 species) and snakes (*N* = 31 species) due to differences in shape and in allometry of testis size and SSD between groups. Pagel's *λ* is given with superscripts corresponding to *P*‐values testing significance using log‐likelihood ratio tests against models of *λ *= 0 and *λ *= 1, respectively

Taxon	Trait	Partial slope ± SE	df	*t*	*P*	Partial *r* (95% CI)	Pagel's *λ*
Lizards	Log_10_ SSD	−0.764 ± 0.226	117	−3.369	<0.001	−0.293 (−5.367, −1.356)	0.352 ^<0.001, <0.001^
	Log_10_ SVL	2.221 ± 0.279	117	7.941	<0.001	0.586 (5.725, 10.129)	0.167 ^0.067, 1^
Snakes	Log_10_ SSD	−0.534 ± 2.423	28	−2.204	0.035	−0.692 (−7.677, −2.946)	<0.001 ^1, <0.001^
	Log_10_ SVL	3.277 ± 0.915	28	3.578	0.001	0.507 (1.132, 5.376)	0.506 ^0.545, 1^

## Results

### Conventional analyses

**Figure 1:**
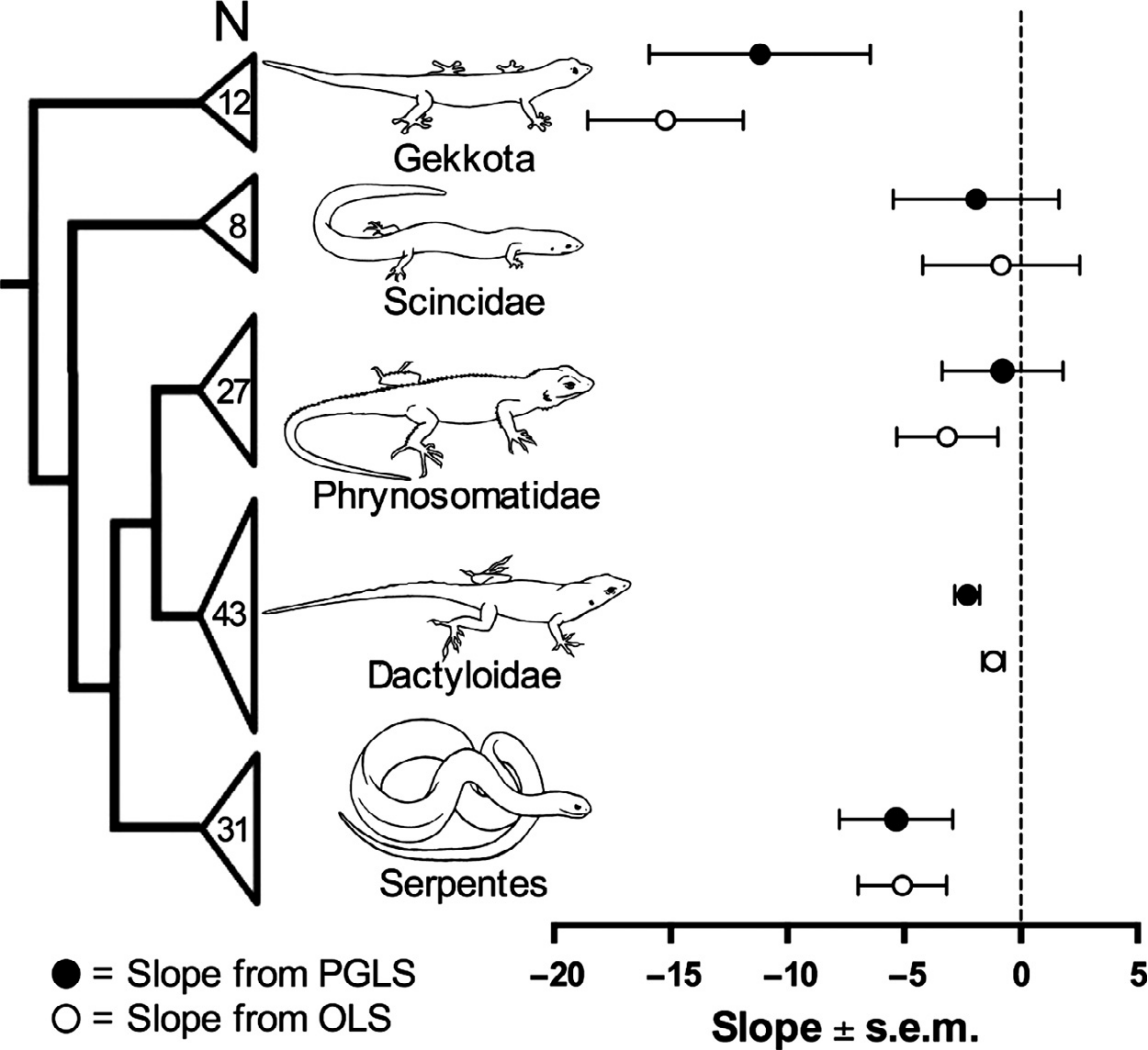
Partial slopes (±SE) from phylogenetic generalized least squares (PGLS, black) and ordinary least squares (OLS, white) regressions of log_10_ testis size on log_10_ sexual size dimorphism (SSD) with log_10_ snout‐vent length (SVL) as a covariate. We conducted separate regressions for all lineages represented by ≥8 species, with sample sizes for each lineage shown at the tips of the phylogeny.

We found a significant negative relationship between log_10_ testis size and log_10_ SSD in lizards (partial *r *=* *−0.149, *P *=* *0.002) and in snakes (partial *r *=* *−0.449, *P *=* *0.012) with log_10_ SVL as a covariate (lizards: partial *r *=* *0.696, *P *<* *0.001; snakes: partial *r *=* *0.592, *P *<* *0.001). We also found a significant negative relationship between log_10_ testis size and log_10_ SSD within the lizard lineages Gekkota (partial *r *=* *−0.835, *P *=* *0.001) and Dactyloidae (partial *r *=* *−0.353, *P *=* *0.021), as well as negative (but nonsignificant) relationships within Scincidae (partial *r *= −0.110, *P *=* *0.813) and Phrynosomatidae (partial *r* = −0.282, *P *=* *0.161; Fig. 1).

### Phylogenetic analyses

**Figure 2:**
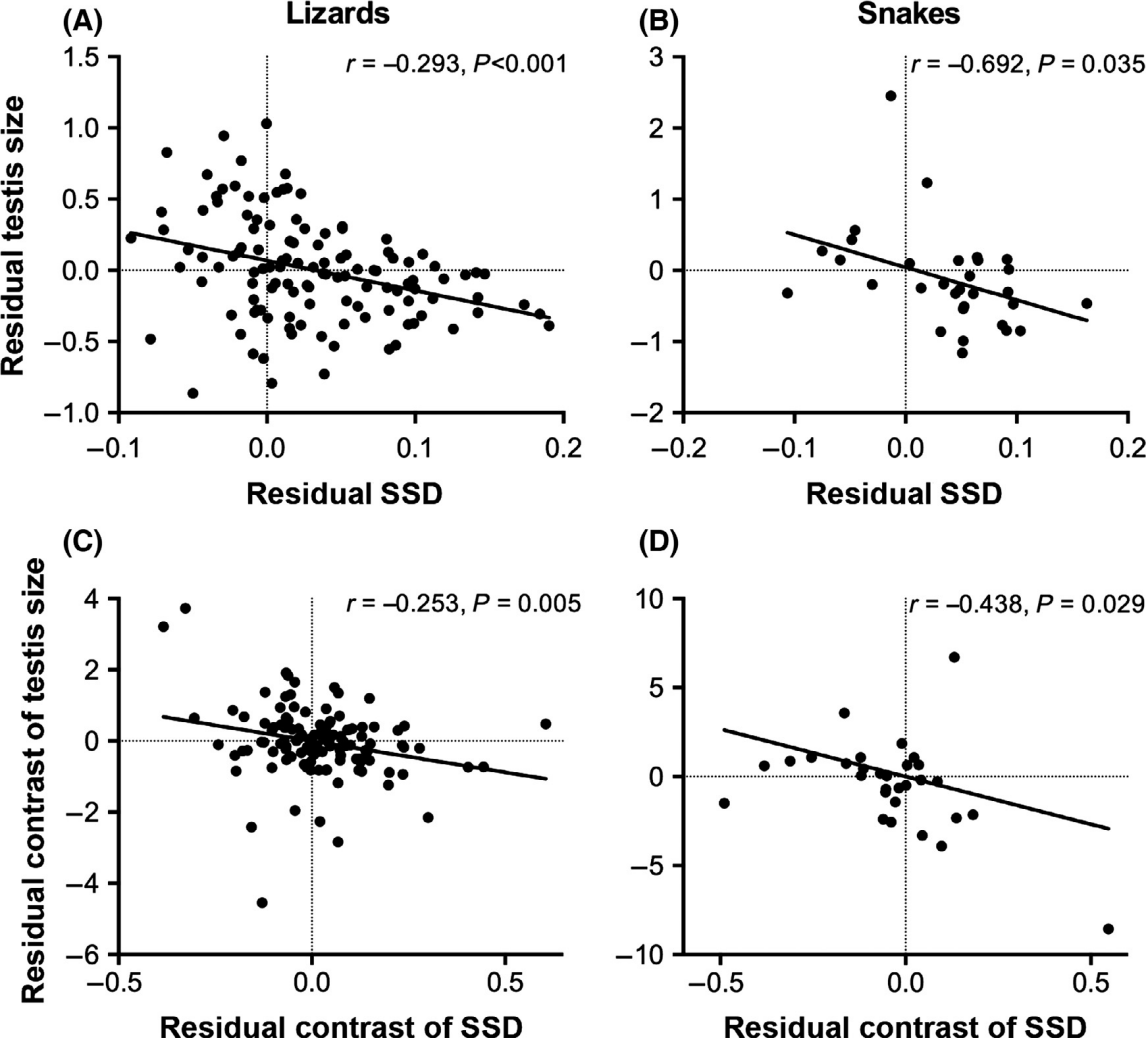
(A, B) Relationships between phylogenetically adjusted and size‐corrected measures of testis size and sexual size dimorphism (SSD) across (A) 120 lizard species and (B) 31 snake species. Residuals were obtained from phylogenetic generalized least squares (PGLS) regression of log_10_ testis size on log_10_ SVL (residual testis size) and PGLS regression of log_10_ SSD on log_10_ SVL (residual SSD). These residuals were used to visualize the covariance between testis size and SSD after accounting for phylogeny and allometry, but actual inferences (and reported partial *r‐* and *P*‐values) are based on multiple regressions using PGLS with a size covariate. (C, D) Relationships between size‐corrected residuals obtained from regression (through the origin) of phylogenetically independent contrasts for log_10_ SSD regressed on independent contrasts for log_10_ SVL (residual contrasts of SSD) and of independent contrasts for log_10_ testis size regressed on independent contrasts for log_10_ SVL (residual contrasts of testis size). As above, relationships are shown separately for (C) lizards (*N* = 119 contrasts) and (D) snakes (*N* = 30 contrasts).

We detected significant phylogenetic signal in log_10_ SSD (Blomberg's *K *=* *0.299, *P *=* *0.001; Pagel's *λ *= 0.352, *P < *0.001, Table 1) and in log_10_ testis size (*K *=* *0.291, *P *=* *0.001; *λ *= 0.167, *P *=* *0.002, Table 1) in lizards. These *K* and *λ* values indicate that related species tend to be more similar than expected by chance, but that variance within clades is greater than predicted under Brownian motion (Pagel 1999; Blomberg et al. 2003). We found similar patterns in snakes, although estimates of *K* and *λ* were not statistically distinguishable from zero (SSD: *K *=* *0.294, *P *=* *0.305; *λ *< 0.001, *P *=* *1; testis size: *K *=* *0.238, *P *=* *0.865; *λ *= 0.506, *P *=* *0.545). When accounting for phylogenetic relationships using PGLS regressions, we recovered the same negative relationships between log_10_ testis size and log_10_ SSD that we detected using conventional analyses in both lizards and snakes (Fig. 2A and B; Table 1). In lizards, these results are robust to the choice between Brownian motion (Table 1) and Ornstein–Uhlenbeck models of character evolution (Table S3). At a finer taxonomic scale, we consistently found negative (although not always significant) relationships between log_10_ testis size and log_10_ SSD within each of the squamate lineages with sufficient representation (*N *≥* *8 species) for separate analyses (Fig. 1; Table S4). We also found that SSD was negatively correlated with testis size after correcting for phylogeny and allometry using phylogenetically independent contrasts in lizards (*r *=* *−0.253, *P *=* *0.005, Fig. 2C) and snakes (*r *=* *−0.438, *P *=* *0.015, Fig. 2D).

## Discussion

We found that the evolution of male‐biased sexual size dimorphism (SSD) in lizards and snakes is consistently associated with a reduction in relative testis size (Table 1, Figs. 1, 2). This negative relationship between the phenotypic targets of pre‐ and postcopulatory sexual selection is consistent across major squamate lineages (Fig. 1) and is similar to macroevolutionary patterns observed in taxa as disparate as voles (Heske and Ostfeld 1990), primates (Lüpold et al. 2014; Dunn et al. 2015), pinnipeds (Fitzpatrick et al. 2012), cetaceans (Dines et al. 2015) and acanthocephalan worms (Poulin and Morand 2000). Notably, the negative correlations that we observed have regression and correlation coefficients near the high end of those reported for most other lineages (Poulin and Morand 2000; Fitzpatrick et al. 2012; Lüpold et al. 2014; Dines et al. 2015). These negative interspecific correlations mirror the trade‐off between body size (or weaponry) and relative testis size that is often observed within species (Moczek and Nijhout 2004; Simmons and Emlen 2006; Kelly 2008; Parker and Pizzari 2010; Yamane et al. 2010; Somjee et al. 2015). However, our results stand in contrast to other comparative studies that have documented non‐significant or even positive interspecific correlations between the targets of pre‐ and postcopulatory sexual selection in bushcrickets (Wedell 1993), ungulates (Ferrandiz‐Rovira et al. 2014; Lüpold et al. 2014), and a variety of other vertebrate and invertebrate taxa (Lüpold et al. 2014). Below, we consider the mating systems of squamate reptiles in light of current theory on the relationship between pre‐ and postcopulatory sexual selection (Parker et al. 2013; Lüpold et al. 2014) and discuss the potential mechanisms structuring the pattern of correlated evolution between SSD and testis size that we have documented in this group.

Recent theory predicts that the interspecific relationship between traits subject to pre‐ and postcopulatory selection should vary depending on the marginal fitness gains associated with increased investment in either type of sexually selected trait (Parker et al. 2013). In particular, male–male contest competition, and the extent to which it results in reproductive monopolization of females, is predicted to be of primary importance. This theory has been tested across several taxonomic groups, and those with higher rates of male–male contest competition and female monopolization (as defined by the percentage of species within a taxonomic group that exhibit female‐defense polygyny) show a stronger negative relationship between SSD or weaponry and testis size (Lüpold et al. 2014). Extrapolating this model to squamate reptiles would imply that strong precopulatory selection for large male body size often results in increased monopolization of females and reduced opportunity for postcopulatory selection. Generally, squamates appear to fit the assumptions of this model, as many snakes and lizards exhibit overt male–male combat, female‐defense territorial polygyny, and mate‐guarding behavior (Stamps 1983; Carothers 1984; Shine 1994). However, multiple paternity occurs in all squamate species that have been studied to date (Uller and Olsson 2008), so the extent to which postcopulatory selection is actually reduced by mate guarding and territory defense is generally uncertain in this lineage. For example, some territorial species exhibit high levels of multiple paternity (e.g., *Anolis sagrei*, Calsbeek et al. 2007), whereas others exhibit low levels of multiple paternity (e.g., *Sceloporus undulatus*, Haenel et al. 2003). Although both species follow the expected pattern ranging from male‐biased SSD and small testis size (*A. sagrei*) to female‐biased SSD and large testis size (*S. undulatus*), this pattern occurs in the absence of categorical variation in territoriality and opposite what would be predicted from the apparent opportunity for postcopulatory sexual selection based on paternity analyses. As such, it is difficult to assess whether the negative correlations that we have documented between SSD and testis size occur for the reasons envisioned by recent theory (Parker et al. 2013; Lüpold et al. 2014). Future studies that partition the opportunities for pre‐ and postcopulatory sexual selection to compare them between related species with contrasting patterns of SSD and testis size could be extremely informative in this regard, and squamates offer a variety of lineages in which mating systems or traits such as sexual size dimorphism vary considerably (e.g., Scincidae, Phrynosomatidae).

Several proximate and ultimate factors may interact to produce the negative correlations that we observed between targets of pre‐ and postcopulatory sexual selection. Ultimately, the sequential nature of these two episodes of selection means that, when precopulatory selection is strong and results in reproductive monopolization of females, the opportunity for postcopulatory selection and sperm competition should be reduced (Preston et al. 2003; Hunt et al. 2009; Fitzpatrick et al. 2012; South and Lewis 2012; Parker et al. 2013). Conversely, when precopulatory selection on males is relaxed, females may re‐mate more frequently, allowing postcopulatory selection to occur (Kvarnemo and Simmons 2013). This does not imply that postcopulatory selection will always be high when precopulatory selection is low nor does it mean that strong precopulatory selection will necessarily preclude postcopulatory selection, particularly when females can re‐mate frequently despite male–male contest competition. As such, the tendency for SSD and testis size to covary negatively despite the presumably imperfect relationship between the strength of pre‐ and postcopulatory selection suggests that proximate energetic constraints on the expression of these traits may further help to structure the negative relationships that we observed. When precopulatory selection is strong, males typically allocate more resources to body size and weaponry, whereas in species where postcopulatory selection is strong, males often invest primarily in traits that improve their success in sperm competition (Moczek and Nijhout 2004; Simmons and Emlen 2006; Kelly 2008; Parker and Pizzari 2010; Yamane et al. 2010; Somjee et al. 2015). Evidence for the latter is limited in squamates, although sperm production is energetically costly in snakes and lizards (Olsson et al. 1997; Kahrl and Cox 2015). Thus, energy allocation trade‐offs among sexually selected traits are likely to act in concert with an inherent covariance in the opportunity for pre‐ and postcopulatory selection to shape the correlated evolution of traits such as body size, weaponry, and testis size.

## Conflict of Interest

The authors declare that they have no conflict of interest.

## Supporting information


**Appendix S1**. Data from all 151 squamate species included in this study.
**Appendix S2.** Details on methodology, date and location of collection for previously unpublished data.
**Table S3.** Summary of results from phylogenetic generalized least squares (PGLS) regression of log_10_ testis size on log_10_ sexual size dimorphism (SSD) with log_10_ mean male body size (snout‐vent length, SVL) as a covariate using an Ornstien‐Uhlenbeck model of character evolution.
**Table S4.** Summary of results from phylogenetic generalized least squares (PGLS) regression of log_10_ testis size on log_10_ sexual size dimorphism (SSD) with log_10_ mean male body size (snout‐vent length, SVL) as a covariate.
**Appendix S5.** Literature sources for data used in this study, as cited in Appendix S1.
