# Supplementary material for: Correlated evolution between targets of pre‐ and postcopulatory sexual selection across squamate reptiles
Source: Ecol Evol. 2016 Aug 18;6(18):6452–9. doi: 10.1002/ece3.2344 (PMC5058519; doi:10.1002/ece3.2344)
Supplement: Supplementary file 1 — Appendix S1. Data from all 151 squamate species included in this study. Appendix S2. Details on methodology, date and location of collection for previously unpublished data. Table S3. Summary of results from phylogenetic generalized least squares (PGLS) regression of log10 testis size on log10 sexual size dimorphism (SSD) with log10 mean male body size (snout‐vent length, SVL) as a covariate using an Ornstien‐Uhlenbeck model of character evolution. Table S4. Summary of results from phylogenetic generalized least squares (PGLS) regression of log10 testis size on log10 sexual size dimorphism (SSD) with log10 mean male body size (snout‐vent length, SVL) as a covariate. Appendix S5. Literature sources for data used in this study, as cited in Appendix S1. [file ECE3-6-6452-s001.docx]

**Appendix S1**. Data from all 151 squamate species included in this study. Sexual size dimorphism (SSD) is calculated as the mean SVL of the larger sex divided by the mean SVL of the smaller sex, expressed as a negative value when females are the larger sex and as a positive value when males are the larger sex. Details on previously unpublished data collected for this study are provided in Appendix S2. Full citations for literature sources are provided in Appendix S5.

| **Species** | **SSD** | **Mean SVL (M)** | **Mean SVL (F)** | **Mean testis size (mm^3^)** | **Number (M, F)** | **Family** | **Source** |
| --- | --- | --- | --- | --- | --- | --- | --- |
| **Lizards** |  |  |  |  |  |  |  |
| *Ameiva ameiva* | 1.022 | 117.8 | 115.3 | 221.6 | 18, 11 | Teiidae | Vitt unpublished |
| *Amphibolurus muricatus* | 1.013 | 91.7 | 90.5 | 47.0 | 87, 69 | Agamidae | Harlow & Taylor 2000 |
| *Anolis aliniger* | 1.169 | 51.7 | 44.2 | 40.4 | 11, 8 | Dactyloidae | This study, Appendix S2 |
| *Anolis angusticeps* | 1.190 | 53.5 | 45.0 | 23.1 | 4, 3 | Dactyloidae | This study, Appendix S2 |
| *Anolis auratus* | 1.019 | 43.6 | 42.8 | 21.6 | 110, 122 | Dactyloidae | Sexton et al. 1971 |
| *Anolis bahrucoensis* | 1.149 | 44.7 | 38.9 | 6.048 | 20, 18 | Dactyloidae | This study, Appendix S2 |
| *Anolis barahonae* | -1.053 | 142.4 | 150.0 | 236.5 | 5, 1 | Dactyloidae | This study, Appendix S2 |
| *Anolis brevirostris* | 1.119 | 49.1 | 43.8 | 12.2 | 19, 16 | Dactyloidae | This study, Appendix S2 |
| *Anolis carolinensis* | 1.192 | 62.8 | 52.7 | 32.5 | 69, 60 | Dactyloidae | Dessauer 1955, Ruby 1984, Stamps et al. 1997 |
| *Anolis christophei* | 1.102 | 47.5 | 43.1 | 13.7 | 20, 20 | Dactyloidae | This study, Appendix S2 |
| *Anolis coelestinus* | 1.270 | 68.7 | 54.1 | 24.8 | 20, 10 | Dactyloidae | This study, Appendix S2 |
| *Anolis conspersus* | 1.534 | 63.5 | 41.4 | 18.0 | - | Dactyloidae | Licht & Gorman 1970 |
| *Anolis chlorocyanus* | 1.254 | 73.8 | 58.9 | 48.2 | 19, 11 | Dactyloidae | This study, Appendix S2 |
| *Anolis cristatellus* | 1.365 | 66.3 | 48.6 | 30.9 | 20, 41 | Dactyloidae | This study, Appendix S2 |
| *Anolis cupreus* | 1.068 | 42.7 | 40.0 | 18.0 | - | Dactyloidae | Fleming & Hooker 1975, Stamps et al. 1997 |
| *Anolis cybotes* | 1.170 | 64.2 | 54.9 | 45.0 | 20, 20 | Dactyloidae | This study, Appendix S2 |
| *Anolis distichus* | 1.140 | 51.6 | 45.3 | 15.2 | 29, 25 | Dactyloidae | This study, Appendix S2 |
| *Anolis equestris* | 1.128 | 151.7 | 134.5 | 310.2 | 9, 6 | Dactyloidae | This study, Appendix S2 |
| *Anolis etheridgei* | 1.216 | 40.7 | 33.5 | 11.3 | 12, 10 | Dactyloidae | This study, Appendix S2 |
| *Anolis evermanni* | 1.277 | 60.2 | 47.2 | 42.3 | 20, 14 | Dactyloidae | This study, Appendix S2 |
| *Anolis fuscoauratus* | -1.109 | 41.1 | 45.6 | 24.5 | 8, 78 | Dactyloidae | Vitt unpublished |
| *Anolis grahami* | 1.389 | 63.9 | 46.0 | 42.0 | - | Dactyloidae | Licht & Gorman 1970 |
| *Anolis gundlachi* | 1.443 | 62.0 | 43.0 | 24.2 | 20, 5 | Dactyloidae | This study, Appendix S2 |
| *Anolis insolitus* | -1.026 | 39.0 | 40.0 | 11.5 | 6, 2 | Dactyloidae | This study, Appendix S2 |
| *Anolis longitibialis* | 1.353 | 65.8 | 48.7 | 44.0 | 17, 20 | Dactyloidae | This study, Appendix S2 |
| *Anolis krugi* | 1.302 | 50.0 | 38.4 | 10.5 | 20, 18 | Dactyloidae | This study, Appendix S2 |
| *Anolis limifrons* | 1.012 | 43.6 | 43.1 | 15.7 | 244, 369 | Dactyloidae | Sexton et al. 1971 |
| *Anolis lineatopus* | 1.368 | 62.5 | 45.7 | 41.0 | - | Dactyloidae | Licht & Gorman 1970 |
| *Anolis marcanoi* | 1.085 | 52.0 | 48.0 | 35.7 | 12, 11 | Dactyloidae | This study, Appendix S2 |
| *Anolis occultus* | -1.036 | 38.4 | 39.8 | 12.8 | 5, 10 | Dactyloidae | This study, Appendix S2 |
| *Anolis olssoni* | 1.053 | 43.8 | 41.7 | 6.1 | 20, 15 | Dactyloidae | This study, Appendix S2 |
| *Anolis opalinus* | 1.196 | 48.3 | 40.4 | 28.8 | 101, 148 | Dactyloidae | Floyd & Jenssen 1983, Jenssen 1994 |
| *Anolis ortonii* | 1.045 | 46.8 | 44.8 | 26.8 | 9, 8 | Dactyloidae | Fitch 1976, Vitt unpublished |
| *Anolis poncensis* | 1.186 | 45.6 | 38.5 | 9.4 | 20, 8 | Dactyloidae | This study, Appendix S2 |
| *Anolis porcatus* | 1.521 | 66.6 | 43.8 | 23.8 | 20, 20 | Dactyloidae | This study, Appendix S2 |
| *Anolis pulchellus* | 1.195 | 44.9 | 37.6 | 9.3 | 20, 16 | Dactyloidae | This study, Appendix S2 |
| *Anolis punctatus* | 1.050 | 77.8 | 74.1 | 31.1 | 10, 11 | Dactyloidae | Vitt unpublished |
| *Anolis richardii* | 1.226 | 93.2 | 76.0 | 95.0 | - | Dactyloidae | Licht & Gorman 1970 |
| *Anolis sagrei* | 1.286 | 56.5 | 44.0 | 30.3 | 25, 25 | Dactyloidae | This study, Appendix S2 |
| *Anolis semilineatus* | 1.000 | 44.0 | 44.0 | 8.6 | 5, 2 | Dactyloidae | This study, Appendix S2 |
| *Anolis smaragdinus* | 1.235 | 60.5 | 49.0 | 33.9 | 25, 25 | Dactyloidae | This study, Appendix S2 |
| *Anolis stratulus* | 1.218 | 47.0 | 38.6 | 31.1 | 20, 20 | Dactyloidae | This study, Appendix S2 |
| *Anolis trachyderma* | -1.158 | 46.9 | 54.3 | 19.8 | 38, 26 | Dactyloidae | Vitt unpublished |
| *Anolis trinitatis* | 1.373 | 63.3 | 46.1 | 22.0 | - | Dactyloidae | Licht & Gorman 1970 |
| *Anolis tropidogaster* | -1.002 | 44.9 | 45.0 | 24.5 | 83, 29 | Dactyloidae | Sexton et al. 1971 |
| *Aspidoscelis tigris* | -1.008 | 77.1 | 77.7 | 63.0 | 44, 75 | Teiidae | Vitt & Ohmart 1977 |
| *Barisia imbricata* | 1.000 | 111.8 | 111.8 | 67.0 | 18, 36 | Anguidae | Guillette & Casas-Andreu 1987 |
| *Basiliscus vittatus* | 1.197 | 116.5 | 97.3 | 62.9 | 15, 15 | Corytophanidae | Hirth 1963, This study, Appendix S2 |
| *Callisaurus draconoides* | 1.201 | 88.3 | 73.6 | 65.6 | 16, 10 | Phrynosomatidae | This study, Appendix S2 |
| *Cnemidophrus lemniscatus* | 1.081 | 65.6 | 54.7 | 62.9 | 67, 21 | Teiidae | Vitt & de Carvalho 1995, Vitt et al. 1997, This study, Appendix S2 |
| *Cophosaurus texanus* | 1.199 | 64.1 | 59.3 | 42.6 | 16, 31 | Phrynosomatidae | This study, Appendix S2 |
| *Crotaphytus collaris* | 1.056 | 96.9 | 91.8 | 365.0 | 422, 518 | Iguanidae | Sexton et al. 1992, Trauth 1979 |
| *Ctenophorus pictus* | 1.000 | 54.0 | 54.0 | 150.0 | 69, 45 | Agamidae | Niejalke 2006 |
| *Ctenotus brooksi* | -1.045 | 44.4 | 46.4 | 16.0 | 105, 98 | Scincidae | James 1991a,b,c |
| *Ctenotus leonhardii* | -1.006 | 65.1 | 65.5 | 21.4 | 106, 110 | Scincidae | James 1991a,b,c |
| *Ctenotus pantherinus* | -1.088 | 79.8 | 86.8 | 86.4 | 61,75 | Scincidae | James 1991a,b,c |
| *Ctenotus piankai* | -1.048 | 50.4 | 52.8 | 14.5 | 81,40 | Scincidae | James 1991a,b,c |
| *Ctenotus quattuordecimlineatus* | -1.038 | 60.4 | 62.7 | 10.3 | 309, 247 | Scincidae | James 1991a,b,c |
| *Ctenotus schomburgkii* | -1.076 | 42.1 | 45.3 | 10.1 | 115, 124 | Scincidae | James 1991a,b,c |
| *Dipsosaurus dorsalis* | 1.058 | 127.0 | 120.0 | 250.0 | 377, 200 | Iguanidae | Mayhew 1971, Licht 1973 |
| *Gambelia sila* | 1.088 | 106.1 | 97.5 | 48.7 | - | Crotaphytidae | Tollestrup 1982 |
| *Gambelia wislizenii* | -1.142 | 104.6 | 119.5 | 39.8 | - | Crotaphytidae | Tollestrup 1982 |
| *Gekko gecko* | 1.087 | 148.6 | 136.7 | 59.0 | 68, 39 | Gekkonidae | Aowphol et al. 2006, This study, Appendix S2 |
| *Gonatodes concinnatus* | 1.010 | 42.4 | 42.0 | 11.3 | 8, 12 | Sphaerodactylidae | Fitch 1981, Duellman unpublished data, Vitt unpublished data |
| *Gonatodes humeralis* | 1.014 | 36.2 | 35.7 | 2.6 | 19, 28 | Sphaerodactylidae | Vitt unpublished |
| *Hemidactylus mabouia* | 1.003 | 63.6 | 63.4 | 35.8 | 12, 14 | Gekkonidae | Vitt unpublished |
| *Hemidactylus palaichthus* | -1.004 | 54.8 | 55.0 | 38.1 | 13, 18 | Gekkonidae | Vitt unpublished |
| *Kentropyx calcarata* | -1.012 | 79.8 | 80.8 | 231.4 | - | Teiidae | Vitt unpublished; Vitt 1991 |
| *Laudakia stellio* | 1.113 | 118.0 | 106.0 | 350.0 | 66, 77 | Agamidae | Childress 1970 |
| *Leiocephalus carinatus* | 1.283 | 106.0 | 82.6 | 36.7 | 3, 8 | Leiocephalidae | This study, Appendix S2 |
| *Leiocephalus barahonensis* | 1.240 | 77.5 | 62.5 | 47.1 | 2, 6 | Leiocephalidae | This study, Appendix S2 |
| *Lepidophyma sylvaticum* | -1.013 | 75.0 | 76.0 | 50.0 | 44, 57 | Xantusiidae | Ramírez-Bautista et al. 2008 |
| *Mabuya nigropunctata* | 1.068 | 87.8 | 82.2 | 30.3 | 15, 12 | Scincidae | Vitt & Blackburn 1991, Vitt unpublished |
| *Ouroborus cataphractus* | 1.009 | 107.0 | 106.0 | 45.0 | 70, 52 | Cordylidae | Flemming & Mouton 2002 |
| *Phrynosoma cornutum* | -1.069 | 69.6 | 74.4 | 466.5 | 4, 2 | Phrynosomatidae | This study, Appendix S2, Fitch 1981 |
| *Phrynosoma modestum* | -1.125 | 55.8 | 62.8 | 158.9 | 4, 2 | Phrynosomatidae | This study, Appendix S2, Fitch 1981 |
| *Phrynosoma solare* | -1.133 | 90.0 | 102.0 | 598.0 | 23, 23 | Phrynosomatidae | Parker 1971, Sherbrooke unpublished |
| *Phyllodactylus lanei* | 1.040 | 68.1 | 65.5 | 78.9 | 199, 144 | Phyllodactylus | Ramírez-Sandoval et al. 2006 |
| *Plestiodon copei* | -1.075 | 58.6 | 63.0 | 47.0 | 100, 100 | Scincidae | Ramírez-Bautista et al. 1996 |
| *Plestiodon laticeps* | 1.154 | 109.4 | 94.8 | 240.0 | 164, 97 | Scincidae | Vitt & Cooper 1985a, 1985b |
| *Plica plica* | 1.085 | 118.2 | 108.9 | 163.7 | 5, 10 | Tropiduridae | Vitt 1991, Vitt unpublished |
| *Plica umbra* | -1.053 | 84.5 | 89.0 | 82.3 | 14, 7 | Tropiduridae | Vitt et al. 1997, Vitt unpublished |
| *Podarcis siculus* | 1.118 | 62.6 | 56.0 | 55.0 | 31, 42 | Lacertidae | Fitch 1981, Nevo et al. 1972 |
| *Pseudocordylus melanotus* | 1.103 | 118.3 | 107.3 | 248.0 | 83, 89 | Cordylidae | Flemming 1993a, 1993b |
| *Sceloporus clarkii* | 1.079 | 84.9 | 78.7 | 273.4 | 6, 3 | Phrynosomatidae | This study, Appendix S2 |
| *Sceloporus graciosus* | -1.035 | 52.1 | 53.9 | 315.0 | 85, 76 | Phrynosomatidae | Jameson 1974, Fitch 1978 |
| *Sceloporus grammicus* | 1.040 | 51.2 | 49.3 | 92.0 | 23, 32 | Phrynosomatidae | Fitch 1978, Guillette & Bearce 1986 |
| *Sceloporus horridus* | -1.099 | 83.2 | 91.4 | 10.4 | 65, 37 | Phrynosomatidae | Valdez-Gonzalez & Ramirez-Bautista 2002 |
| *Sceloporus jarrovii* | 1.068 | 88.2 | 82.6 | 129.1 | 6, 5 | Phrynosomatidae | Cox and John-Alder 2007, This study, Appendix S2 |
| *Sceloporus magister* | 1.146 | 102.5 | 89.4 | 108.6 | 17, 24 | Phrynosomatidae | This study, Appendix S2 |
| *Sceloporus malachiticus* | 1.048 | 79.1 | 75.5 | 259.0 | 146, 208 | Phrynosomatidae | Marion & Sexton 1971, Fitch 1978 |
| *Sceloporus occidentalis* | 1.036 | 75.3 | 72.8 | 231.0 | 97, 46 | Phrynosomatidae | Fitch 1978, Wilhoft & Quay 1961 |
| *Sceloporus olivaceus* | -1.135 | 93.1 | 105.7 | 183.5 | 11, 11 | Phrynosomatidae | This study, Appendix S2 |
| *Sceloporus orcutti* | 1.109 | 102.0 | 92.0 | 225.0 | 117, 77 | Phrynosomatidae | Mayhew 1963 |
| *Sceloporus scalaris* | -1.077 | 47.5 | 51.3 | 60.0 | 45, 203 | Phrynosomatidae | Newlin 1976 |
| *Sceloporus spinosus* | 1.039 | 90.4 | 87.0 | 14.5 | 73, 23 | Phrynosomatidae | Valdéz-González & Ramírez-Bautista 2002 |
| *Sceloporus undulatus* | -1.054 | 62.0 | 65.4 | 247.0 | 85, 79 | Phrynosomatidae | Cooper & Vitt 1989, Marion 1982 |
| *Sceloporus utiformis* | 1.079 | 64.4 | 59.7 | 15.6 | 9, 10 | Phrynosomatidae | Fitch 1978, Ramírez-Bautista & Gutiérrez-Mayén 2003 |
| *Sceloporus virgatus* | -1.108 | 56.4 | 62.5 | 114.7 | 12, 11 | Phrynosomatidae | Rose 1981, This study, Appendix S2 |
| *Sceloporus woodi* | -1.061 | 47.6 | 50.5 | 55.7 | 150, 170 | Phrynosomatidae | Jackson & Telford 1974 |
| *Strophurus ciliaris* | -1.083 | 67.4 | 73.0 | 150.0 | 64, 82 | Diplodactylidae | How et al. 1986 |
| *Strophurus elderi* | -1.059 | 40.6 | 43.0 | 37.0 | 72, 81 | Diplodactylidae | How et al. 1986 |
| *Strophurus intermedius* | -1.092 | 58.7 | 64.1 | 140.0 | 33, 43 | Diplodactylidae | How et al. 1986 |
| *Strophurus rankini* | -1.053 | 53.1 | 55.9 | 60.0 | 20, 23 | Diplodactylidae | How et al. 1986 |
| *Strophurus spinigerus* | -1.077 | 56.1 | 60.4 | 134.0 | 77, 101 | Diplodactylidae | How et al. 1986 |
| *Strophurus strophurus* | -1.127 | 58.1 | 65.5 | 97.0 | 53, 73 | Diplodactylidae | How et al. 1986 |
| *Takydromus hsuehshanensis* | -1.012 | 60.5 | 61.2 | 42.0 | 78, 105 | Lacertidae | Huang 1998 |
| *Takydromus sylvaticus* | -1.053 | 53.0 | 55.8 | 19.1 | 5, 4 | Lacertidae | Tang 2007 |
| *Trachylepis capensis* | -1.223 | 75.4 | 92.3 | 105.0 | 90, 71 | Scincidae | Flemming 1994 |
| *Tropidurus hispidus* | 1.290 | 93.5 | 72.5 | 58.6 | 39, 42 | Tropiduridae | Vitt unpublished; Vitt 1993 |
| *Tropidurus itambere* | 1.128 | 75.9 | 67.3 | 56.2 | 66, 60 | Tropiduridae | Van Sluys 1993a,b, 1998, 2000 |
| *Tropidurus torquatus* | 1.168 | 96.1 | 82.3 | 200.0 | 224, 233 | Tropiduridae | Vitt & Goldberg 1983 |
| *Uma inornata* | 1.259 | 102.0 | 81.0 | 190.0 | 191, 213 | Phrynosomatidae | Mayhew 1965 |
| *Uma notate* | 1.263 | 96.0 | 76.0 | 123.3 | 214, 270 | Phrynosomatidae | Mayhew 1966a |
| *Uma scoparia* | 1.169 | 97.0 | 83.0 | 210.0 | 248, 236 | Phrynosomatidae | Mayhew 1966b |
| *Uranoscodon superciliosus* | -1.127 | 101.2 | 114.1 | 289.5 | 22, 15 | Tropiduridae | Vitt unpublished |
| *Urosaurus graciosus* | 1.034 | 54.1 | 52.3 | 25.0 | 227, 195 | Phrynosomatidae | Vitt et al. 1978 |
| *Urosaurus ornatus* | 1.122 | 55.6 | 49.6 | 34.3 | 17, 25 | Phrynosomatidae | This study, Appendix S2 |
| *Uta stansburiana* | 1.100 | 54.4 | 49.5 | 19.6 | 17, 14 | Phrynosomatidae | This study, Appendix S2 |
| *Xantusia vigilis* | -1.079 | 38.0 | 41.0 | 16.0 | - | Xantusiidae | Miller 1951, Licht 1973 |
| **Snakes** |  |  |  |  |  |  |  |
| *Acrochordus arafurae* | -1.286 | 1050 | 1350 | 5654.0 | 217, 331 | Acrochordidae | Shine 1986 |
| *Acrochordus granulatus* | -1.077 | 648 | 698 | 600.0 | 168, 147 | Acrochordidae | Gorman et al. 1981 |
| *Agkistrodon piscivorus* | 1.100 | 767 | 697 | 470.0 | 93, 49 | Viperidae | Scott et al. 1995 |
| *Austrelaps superbus* | 1.085 | 766 | 706 | 200.0 | 110, 27 | Elapidae | Shine 1977a,b, Shine 1978 |
| *Boa constrictor* | -1.139 | 1800 | 2050 | 15840.0 | 56, 50 | Boidae | Bertona & Chiaraviglio 2003 |
| *Boiga irregularis* | 1.213 | 1308 | 1078 | 735.0 | 455, 327 | Colubridae | Savidge et al. 2007 |
| *Bothrops asper* | -1.126 | 1263 | 1422 | 3000.0 | 64, 83 | Viperidae | Solorzano & Cerdas 1989 |
| *Cerastes vipera* | -1.102 | 235 | 259 | 80.0 | 165, 165 | Viperidae | Sivan et al. 2012 |
| *Cerberus rynchops* | -1.041 | 511 | 532 | 720.0 | 203, 179 | Colubridae | Gorman et al. 1981 |
| *Crotalus adamanteus* | 1.003 | 1107 | 1104 | 474.0 | 32, 31 | Viperidae | Steen et al. 2007, Hoss et al. 2011 |
| *Crotalus oreganus* | 1.332 | 986 | 740 | 890.0 | 39, 19 | Viperidae | Lind et al. 2010 |
| *Cylindrophis ruffus* | -1.042 | 686 | 715 | 508.0 | 48, 88 | Cylindrophiidae | Brooks et al. 2009 |
| *Enhydrina schistosa* | -1.083 | 782 | 847 | 25000.0 | 38, 13 | Elapidae | Voris & Jayne 1979 |
| *Enhydris bocourti* | -1.077 | 532 | 573 | 787.6 | 68, 68 | Colubridae | Brooks et al. 2009 |
| *Enhydris enhydris* | -1.063 | 475 | 505 | 203.8 | 2341, 1754 | Colubridae | Brooks et al. 2009, Karns et al. 2005 |
| *Enhydris longicauda* | -1.106 | 442 | 489 | 195.6 | 720, 906 | Colubridae | Brooks et al. 2009 |
| *Erpeton tentaculatum* | -1.092 | 459 | 501 | 114.5 | 443, 424 | Colubridae | Brooks et al. 2009 |
| *Hemiaspis signata* | -1.005 | 431 | 433 | 170.0 | 53, 15 | Elapidae | Shine 1977a,b, Shine 1978 |
| *Homalopsis buccata* | 1.000 | 740 | 740 | 1074.1 | 707, 874 | Colubridae | Brooks et al. 2009 |
| *Laticauda colubrina* | -1.419 | 884.5 | 1255 | 950.0 | 158, 73 | Elapidae | Gorman et al. 1981 |
| *Micrurus fulvius* | -1.329 | 547 | 727 | 2240.0 | 51, 27 | Elapidae | Jackson & Franz 1981 |
| *Nerodia taxispilota* | -1.214 | 608 | 738 | 225000.0 | 70, 54 | Colubridae | White et al. 1982 |
| *Notechis scutatus* | 1.002 | 810 | 808 | 500.0 | 174, 32 | Elapidae | Shine 1977a,b, Shine 1978 |
| *Pseudechis porphyriacus* | 1.052 | 1116 | 1061 | 360.0 | 225, 55 | Elapidae | Shine 1977a,b, Shine 1978 |
| *Pseudonaja textilis* | 1.068 | 1250 | 1170 | 320.0 | - | Elapidae | Shine 1977a,b, Whitaker & Shine 2003 |
| *Sibynomorphus mikanii* | -1.241 | 352 | 437 | 117.6 | 92, 52 | Colubridae | Rojas et al. 2013, Pizzatto et al. 2008 |
| *Thamnophis elegans* | -1.081 | 405 | 438 | 451.5 | 22, 20 | Colubridae | Fox 1954 |
| *Thamnophis melanogaster* | 1.021 | 546 | 535 | 103.5 | 15, 28 | Colubridae | Garstka & Crews 1982 |
| *Thamnophis sirtalis* | -1.012 | 417 | 422 | 451.5 | 68, 92 | Colubridae | White & Kolb 1974 |
| *Tropidoclonion lineatum* | -1.565 | 200 | 313 | 141.2 | 65, 100 | Colubridae | Krohmer & Aldridge 1985a, 1985b |
| *Xenochrophis piscator* | -1.202 | 504 | 606 | 628.9 | 97,135 | Colubridae | Brooks et al. 2009 |

**Appendix S2.** Details on methodology, date and location of collection for previously unpublished data. We collected all data during the peak of the breeding season (May-June) to ensure that testes were at their maximum size. We captured lizards using nooses and measured them for mass and snout-vent length. We checked each male for sexual maturity by collecting a sperm sample. Any male who did not produce sperm within one day of capture was excluded from the dataset. Testis size for each species was measured by surgical laparotomy or dissection. Prior to laparotomy, each lizard received a 2-4 μl intraperitoneal injection of 0.25% bupivicaine (Auromedics, Dayton, NJ) as a local anesthetic and analgesic. We then cooled the lizards at

-20**°**F for 5-10 minutes and immobilized them on a slightly thawed ice pack during surgery. We made a small ventral incision and externalized the left testis to measure its length and width using a dial caliper, then returned the testis to the abdomen and closed the incision with Nexaband surgical glue (Veterinary Products Laboratories, Phoenix, AZ). We held each individual in separate container overnight to allow them to recover, then released them to their capture locations. Species from Arizona were collected under scientific collecting permit SP673841 issued to Dr. Robert M. Cox by the Arizona Game and Fish Department. Species from Puerto Rico were collected under permits 2014-IC-045 and 2014-IC-029 issued to Dr. Michele Johnson by el Departmento de Recursos Naturales y Ambientales. Species from the Dominican Republic were collected under permits issued to Dr. Michele Johnson by el Ministerio de Medio Abiente y Recursos Naturales. Species from Texas were collected under permit SPR 0814-159 issued to Corey E. Roelke by the Texas Parks and Wildlife Department. Species from Dade County, Florida were collected under permit 213 issued to Ariel F. Kahrl by Miami-Dade County Parks and Recreation.

| **Species** | **Testes measurements** | **Date** | **GPS** |
| --- | --- | --- | --- |
| *Anolis aliniger* | Dissection | June 2015 | N 19° 01' 57.52" W 70° 32' 35.50" |
| *Anolis angusticeps* | Dissection | June 2013 | N 23° 30' 23.17" W 75° 45' 57.53" |
| *Anolis bahorucoensis* | Dissection | June 2015 | N 18° 07' 36.66" W 71° 16' 06.67" |
| *Anolis barahonae* | Dissection | June 2015 | N 18° 05' 59.92" W 71° 15' 14.42" |
| *Anolis brevirostris* | Dissection | June 2015 | N 18° 03' 29.24" W 71° 06' 46.51" |
| *Anolis christophei* | Dissection | June 2015 | N 19° 01' 57.52" W 70° 32' 35.50" |
| *Anolis coelestinus* | Dissection | June 2015 | N 18° 03' 29.24" W 71° 06' 46.51" |
| *Anolis chlorocyanus* | Dissection | June 2015 | N 18° 31' 38.92" W 70° 30' 30.43" |
| *Anolis cristatellus* | Dissection | June 2014 | N 18° 20' 32.28" W 65° 49' 33.72" |
| *Anolis cybotes* | Dissection | June 2015 | N 18° 03' 29.24" W 71° 06' 46.51" |
| *Anolis distichus* | Dissection | June 2013 | N 23° 30' 23.17" W 75° 45' 57.53" |
| *Anolis equestris* | Dissection | May 2014 | N 25° 36' 56.36" W 80° 18' 24.19" |
| *Anolis etheridgei* | Dissection | June 2015 | N 19° 01' 57.52" W 70° 32' 35.50" |
| *Anolis evermanni* | Dissection | June 2014 | N 18° 20' 32.28" W 65° 49' 33.72" |
| *Anolis gundlachi* | Dissection | June 2014 | N 18° 20' 32.28" W 65° 49' 33.72" |
| *Anolis insolitus* | Dissection | June 2015 | N 19° 02' 26.71" W 70° 31' 17.34" |
| *Anolis longitibialis* | Dissection | June 2015 | N 17° 50' 10.36" W 71° 27' 00.09" |
| *Anolis krugi* | Dissection | June 2014 | N 18° 20' 32.28" W 65° 49' 33.72" |
| *Anolis marcanoi* | Dissection | June 2015 | N 18° 24' 21.22" W 70° 25' 02.36" |
| *Anolis occultus* | Dissection | June 2014 | N 18° 27' 09.00" W 66° 35' 49.56" |
| *Anolis olssoni* | Dissection | June 2015 | N 18° 13' 50.80" W 70° 20' 44.15" |
| *Anolis poncensis* | Dissection | June 2014 | N 17° 56' 56.94" W 66° 52' 32.64" |
| *Anolis porcatus* | Dissection | May 2014 | N 25° 42' 28.51" W 80° 09' 27.75" |
| *Anolis pulchellus* | Dissection | June 2014 | N 18° 19' 52.56" W 65° 49' 26.58" |
| *Anolis sagrei* | Dissection | June 2013 | N 23° 30' 23.17" W 75° 45' 57.53" |
| *Anolis semilineatus* | Dissection | June 2015 | N 18° 51' 25.33" W 70° 41 '45.48" |
| *Anolis smaragdinus* | Dissection | June 2013 | N 23° 29' 49.14" W 75° 45' 54.59" |
| *Anolis stratulus* | Dissection | June 2014 | N 18° 20' 32.28" W 65° 49' 33.72" |
| *Basiliscus vittatus* | Dissection | May 2014 | N 25° 40' 42.31" W 80° 16' 25.60" |
| *Callisaurus draconoides* | Laparotomy | May 2014 | N 32° 16' 50.33" W 110° 55' 58.28" |
| *Cnemidophorus lemniscatus* | Dissection | May 2014 | N 25° 47' 34.96" W 80° 12' 48.18" |
| *Cophosaurus texanus* | Dissection | May 2014 | N 32° 20' 22.36" W 110° 54' 34.43" |
| *Gekko gecko* | Dissection | May 2014 | N 25° 36' 55.16" W 80° 18' 23.42" |
| *Leiocephalus carinatus* | Dissection | May 2014 | N 25° 47' 34.96" W 80° 12' 48.18" |
| *Leiocephalus barahonensis* | Dissection | May 2014 | N 17° 50' 10.36" W 71° 27' 00.09" |
| *Phrynosoma cornutum* | Laparotomy | May 2014 | N 31 °54' 50.32" W 109° 08' 29.32" |
| *Phrynosoma modestum* | Laparotomy | May 2014 | N 31 °54' 50.32" W 109° 08' 29.32" |
| *Sceloporus clarkii* | Laparotomy | May 2014 | N 32° 19' 48.41" W 110° 51' 21.71" |
| *Sceloporus jarrovii* | Laparotomy | May 2014 | N 31° 52' 10.95" W 109° 11' 10.20" |
| *Sceloporus magister* | Laparotomy | May 2014 | N 32° 16' 50.33" W 110° 55' 58.28" |
| *Sceloporus olivaceus* | Dissection | July 2014 | N 32° 45' 01.44" W 97° 07' 54.52" |
| *Sceloporus virgatus* | Laparotomy | May 2014 | N 31° 54' 11.36" W 109° 14' 35.39" |
| *Urosaurus ornatus* | Laparotomy | May 2014 | N 32° 16' 50.33" W 110° 55' 58.28" |
| *Uta stansburiana* | Laparotomy | May 2014 | N 32° 16' 50.33" W 110° 55' 58.28" |

**Supplementary Table S3** Summary of results from phylogenetic generalized least squares (PGLS) regression of log_10_ testis size on log_10_ sexual size dimorphism (SSD) with log_10_ mean male body size (snout-vent length, SVL) as a covariate using an Ornstien-Uhlenbeck model of character evolution.

| **Taxon, trait** | **Partial slope ± s.e.** | **df** | **t value** | **P value** | **Partial r, 95% CI (LCL, UCL)** |
| --- | --- | --- | --- | --- | --- |
| **Lizards** |  |  |  |  |  |
| Log10 SSD | -0.764 ± 0.226 | 120 | -3.369 | <0.001* | -0.180, (-3.981, -0.028) |
| Log10 SVL | 2.221 ± 0.279 | 120 | 7.941 | <0.001* | 0.200, (0.253, 4.213) |

**Supplementary Table S4** Summary of results from phylogenetic generalized least squares (PGLS) regression of log_10_ testis size on log_10_ sexual size dimorphism (SSD) with log_10_ mean male body size (snout-vent length, SVL) as a covariate. Analyses were conducted separately for each taxonomic group. Pagel’s λ is given with superscripts corresponding to *P*-values testing significance using log-likelihood ratio tests against models of λ=0, and λ=1, respectively.

| **Taxon, trait** | **Partial slope ± s.e.** | **df** | **t value** | **P value** | **Partial r, 95% CI (LCL, UCL)** | **Pagel’s λ** |
| --- | --- | --- | --- | --- | --- | --- |
| **Dactyloidae** |  |  |  |  |  |  |
| Log10 SSD | -2.289 ± 0.532 | 43 | -4.296 | <0.001* | -0.548, (-6.434, -2.116) | <0.001^1, 0.812^ |
| Log10 SVL | 2.608 ± 0.283 | 43 | 9.213 | <0.001* | 0.814, (6.426, 11.943) | <0.001^1, 1^ |
| **Phrynosomatidae** |  |  |  |  |  |  |
| Log10 SSD | -0.784 ± 2.599 | 27 | -0.301 | 0.765 | -0.057, (-2.259, 1.663) | 0.744 ^0.690, 1^ |
| Log10 SVL | 1.508 ± 0.948 | 27 | 1.591 | 0.124 | 0.292, (-0.427, 3.581) | <0.001 ^1, 1^ |
| **Gekkota** |  |  |  |  |  |  |
| Log10 SSD | -11.173 ± 4.733 | 12 | -2.360 | 0.042* | -0.563, (-4.491, -0.149) | 0.785 ^0.005, 1^ |
| Log10 SVL | 2.271 ± 3.434 | 12 | 3.434 | 0.007* | 0.704, (1.001, 5.770) | <0.001 ^1, 1^ |
| **Scincidae** |  |  |  |  |  |  |
| Log10 SSD | -1.906 ± 3.556 | 8 | -0.536 | 0.614 | -0.186, (-2.496, 1.457) | 0.999 ^0.450, 009^ |
| Log10 SVL | 2.618 ± 1.311 | 8 | 1.996 | 0.102 | 0.576, (-0.236, 4.130) | <0.001 ^1, 0.089^ |

**Appendix S5.** Literature sources for data used in this study, as cited in Appendix S1.

Aowphol, A., Thirakhupt, K., Nabhitabhata, J. & Voris, H.K. 2006. Foraging ecology of the Tokay gecko, *Gekko gecko* in a residential area in Thailand. *Amphibia-Reptilia* **27**:491-503.

Bertona, M. & Chiaraviglio, M. 2003. Reproductive biology, mating aggregations, and sexual dimorphism of the Argentine Boa Constrictor (*Boa constrictor occidentalis*). *J. Herpetol.* **37**:510-516.

Brooks, S.E., Allison, E.H., Gill, J.A. & Reynolds, J.D. 2009. Reproductive and trophic ecology of an assemblage of aquatic and semi-aquatic snakes in Tonle Sap, Cambodia. *Copeia* **2009**:7-20.

Childress, J.R. 1970. Observations on the reproductive cycle of *Agama stellio picea*. *Herpetologica.* **26**:149-155.

Cooper, W.E. & Vitt, L.J. 1989. Sexual dimorphism of head and body size in an iguanid lizard: paradoxical results. *Am. Nat.* **133**:729-735.

Cox, R.M. & John-Alder, H.B. 2007. Growing apart together: the development of contrasting sexual size dimorphisms in sympatric *Sceloporus* lizards. *Herpetologica*. **63**:245-257.

Dessauer, H.C. 1955. Seasonal changes in the gross organ composition of the lizard, *Anolis* *carolinensis*. *J. Exp. Zool.* **128**:1-12.

Fitch, H.S. 1976. Sexual size differences in the mainland anoles. Occas. Pap. Mus. Nat. Hist. Univ. Kans. 50:1–21.

Fitch, H.S. 1978. Sexual size differences in the genus Sceloporus. Uni. Kansas. Sci. Bull. 51:441-461.

Fitch, H.S. 1981. Sexual size differences in reptiles. Mis. Publ. Mus. Nat. Hist. Uni. Kans. 70:1-72.

Fleming, T.H. & Hooker, R.S. 1975. *Anolis cupreus*: the response of a lizard to tropical seasonality. *Ecology*. **56**:1243-1261.

Flemming, A.F. 1993a. The male reproductive cycle of the lizard *Pseudocordylus m. melanotus* (Sauria: Cordylidae). *J. Herpetol.* **27**:473-478.

Flemming, A.F. 1993b. The female reproductive cycle of the lizard *Pseudocordylus m. melanotus* (Sauria: Cordylidae). *J. Herpetol.* **27**:103-107.

Flemming, A.F. 1994. Male and female reproductive cycles of the viviparous lizard, *Mabuya capensis* (Sauria: Scincidae) from South Africa. *J. Herpetol*. **28**:334-341.

Flemming, A.F. & Mouton, P.L.F.N. 2002. Reproduction in a group-living lizard, *Cordylus cataphractus* (Cordylidae), from South Africa*. J. Herpetol*. **36**:691-696.

Floyd, H.B. & Jenssen, T.A. 1983. Food habits of the Jamaican lizard *Anolis opalinus*: resource partitioning and seasonal effects examined. *Copeia* **1983**:319-331.

Fox, W. 1954. Genetic and environmental variation in the timing of the reproductive cycles of male garter snakes. *J. Morphol*. **95**: 415–450.

Garstka, W.R. & Crews, D. 1982. Female control of male reproductive function in a Mexican snake. *Science* **217**:1159-1160.

Gorman, G.C., Licht, P. & McCollum, F. 1981. Annual reproductive patterns in three species of marine snakes from the central Phillippines. *J. Herpetol*. **15**:335-354.

Guillette Jr., L.J. & Bearce, D.A. 1986. The reproductive and fat body cycles of the lizard, *Sceloporus grammicus disparilis*. *Trans. Kans. Acad. Sci*. **89**:31-39.

Guillette Jr., L.J. & Casas-Andreu, G. 1987. The reproductive biology of the high elevation Mexican lizard *Barisia imbricata*. *Herpetologica* **43**:29-38.

Harlow, P.S. & Taylor, J.E. 2000. Reproductive ecology of the jacky dragon (*Amphibolurus muricatus*): an agamid lizard with temperature‐dependent sex determination. *Austral Ecol.***25**:640-652.

Hirth, H.F. 1963. The ecology of two lizards on a tropical beach. *Ecol. Monogr.* **33**:83-112.

How, R.A., Dell, J. & Wellington, B.D. 1986. Comparative biology of eight species of *Diplodactylus* gecko in Western Australia. *Herpetologica* **42**:471-482.

Hoss, S.K., Schuett, G.W., Earley, R.L. & Smith, L.L. 2011. Reproduction in male *Crotalus adamanteus* Beauvois (Eastern diamond-backed rattlesnake): relationship of plasma testosterone to testis and kidney dimensions and the mating season. *Southeast. Nat.***10**:95-108.

Huang, W.S. 1998. Reproductive cycles of the grass lizard, *Takydromus hsuehshanensis*, with comments on reproductive patterns of lizards from the central high elevation area of Taiwan. *Copeia* **1998**:866-873.

Jackson, D.R. & Franz, R. 1981. Ecology of the eastern coral snake (*Micrurus fulvius*) in northern peninsular Florida. *Herpetologica* **37**:213-228.

Jackson, J.F. & Telford Jr., S.R. 1974. Reproductive ecology of the Florida scrub lizard, *Sceloporus woodi*. *Copeia* **1974**:689-694.

James, C.D. 1991. Population dynamics, demography, and life history of sympatric scincid lizards (*Ctenotus*) in central Australia. *Herpetologica* **47**:194-210.

James, C.D. 1991. Annual variation in reproductive cycles of scincid lizards (*Ctenotus*) in central Australia. *Copeia* **1991**:744-760.

James, C.D. 1991. Growth rates and ages at maturity of sympatric scincid lizards (*Ctenotus*) in central Australia. *J. Herpetol.* **25**:284-295.

Jameson Jr, E.W. 1974. Fat and breeding cycles in a montane population of *Sceloporus graciosus. J. Herpetol*. **8**:311-322.

Jenssen, T.A. & Nunez, S.C. 1994. Male and female reproductive cycles of the Jamaican lizard, *Anolis opalinus*. *Copeia* **1994**:767-780.

Karns, D.R., Murphy, J.C., Voris, H.K. & Suddeth, J.S. 2005. Comparison of semi-aquatic snake communities associated with the Khorat Basin, Thailand. *The Natural History Journal of Chulalongkorn University* **5**:73-90.

Krohmer, R.W. & Aldridge, R.D. 1985a. Male reproductive cycle of the lined snake *(Tropidoclonion lineatum). Herpetologica* **41**:33-38.

Krohmer, R.W. & Aldridge, R.D. 1985b. Female reproductive cycle of the lined snake (*Tropidoclonion lineatum*). *Herpetologica* **41**:39-44.

Licht, P. & Gorman, G.C. 1970. Reproductive and fat cycles in Caribbean *Anolis* lizards (Vol. 95). USA: University of California Press.

Licht, P. 1973. Environmental influences on the testis cycles of the lizards *Dipsosaurus dorsalis* and *Xantusia vigilis*. *Comp. Biochem. Phys. A*. **45**:7-20.

Lind, C.M., Husak, J.F., Eikenaar, C., Moore, I.T. & Taylor, E.N. 2010. The relationship between plasma steroid hormone concentrations and the reproductive cycle in the Northern Pacific Rattlesnake, *Crotalus oreganus*. *Gen. Comp. Endocr*.**166**:590-599.

Marion, K.R. & Sexton, O.J. 1971. The reproductive cycle of the lizard *Sceloporus malachiticus* in Costa Rica. *Copeia* **1971**:517-526.

Marion, K.R. 1982. Reproductive cues for gonadal development in temperate reptiles: temperature and photoperiod effects on the testicular cycle of the lizard *Sceloporus undulatus.* *Herpetologica* **38**:26-39.

Mayhew, W.W. 1963. Reproduction in the granite spiny lizard, *Sceloporus orcutti*. *Copeia* **1963**:144-152.

Mayhew, W.W. 1965. Reproduction in the sand-dwelling lizard *Uma inornata*. *Herpetologica* **21**:39-55.

Mayhew, W.W. 1966a. Reproduction in the arenicolous lizard *Uma notata*. *Ecology* **47**:9-18.

Mayhew, W.W. 1966b. Reproduction in the psammophilous lizard *Uma scoparia*. *Copeia* **1966**:114-122.

Mayhew, W.W. 1971. Reproduction in the desert lizard, *Dipsosaurus dorsalis*. *Herpetologica* **27**:57-77.

Nevo, E., Gorman, G.C., Soulé, M., Yang, S.Y., Clover, R. & Jovanović, V. 1972. Competitive exclusion between insular *Lacerta* species (Sauria, Lacertidae). *Oecologia***10**:183-190.

Miller, M.R. 1951. Some aspects of the life history of the yucca night lizard, *Xantusia vigilis.* *Copeia* **1951**:114-120.

Newlin, M.E. 1976. Reproduction in the bunch grass lizard, *Sceloporus scalaris*. *Herpetologica* **32**:171-184.

Niejalke, D.P. 2006. Reproduction by a small agamid lizard, *Ctenophorus pictus*, during contrasting seasons. *Herpetologica* **62**:409-420.

Parker, W.S. 1971. Ecological observations on the regal horned lizard (*Phrynosoma solare*) in Arizona. *Herpetologica* **27**:333-338.

Pizzatto, L., Cantor, M., De Oliveira, J.L., Marques, O.A., Capovilla, V. & Martins, M. 2008. Reproductive ecology of dipsadine snakes, with emphasis on South American species. *Herpetologica* **64**:168-179.

Ramirez-Bautista, A., Guillette Jr., L.J., Gutierrez-Mayen, G. & Uribe-Peña, Z. 1996. Reproductive Biology of the Lizard "*Eumeces Copei*" (Lacertilia: Scincidae) from the Eje Neovolcanico, Mexico. *Southwest. Nat.* **41**:103-110.

Ramírez-Bautista, A. & Gutiérrez-Mayén, G. 2003. Reproductive ecology of *Sceloporus utiformis* (Sauria: Phrynosomatidae) from a tropical dry forest of Mexico. *J. Herpetol.* **37**:1-10.

Ramírez-Bautista, A., Vitt, L.J., Ramírez-Hernández, A., Quijano, F.M. & Smith, G.R. 2008. Reproduction and sexual dimorphism of *Lepidophyma sylvaticum* (Squamata: Xantusiidae), a tropical night lizard from Tlanchinol, Hidalgo, Mexico*. Amphibia-Reptilia***29**:207-216.

Ramírez-Sandoval, E., Ramírez-Bautista, A. & Vitt, L.J. 2006. Reproduction in the lizard *Phyllodactylus lanei* (Squamata: Gekkonidae) from the Pacific Coast of Mexico. *Copeia* **2006**:1-9.

Rojas, C.A., Barros, V.A. & Almeida‐Santos, S.M. 2013. The reproductive cycle of the male sleep snake *Sibynomorphus mikanii* (Schlegel, 1837) from southeastern brazil. *J. Morphol.***274**:215-228.

Rose, B. 1981. Factors affecting activity in *Sceloporus virgatus*. *Ecology* **62**:706-716.

Ruby, D.E. 1984. Male breeding success and differential access to females in *Anolis carolinensis.* *Herpetologica* **40**:272-280.

Savidge, J.A., Qualls, F.J. & Rodda, G.H. 2007. Reproductive biology of the brown tree snake, *Boiga irregularis* (Reptilia: Colubridae), during colonization of Guam and comparison with that in their native range 1. *Pac. Sci.***61**:191-199.

Scott, D.E., Fischer, R.U., Congdon, J.D. & Busa, S.A. 1995. Whole body lipid dynamics and reproduction in the eastern cottonmouth, *Agkistrodon piscivorus*. *Herpetologica* **51**:472-487.

Sexton, O.J., Ortleb, E.P., Hathaway, L.M., Ballinger, R.E. & Licht, P. 1971. Reproductive cycles of three species of anoline lizards from the Isthmus of Panama. *Ecology* **52**:202-215.

Sexton, O.J., Andrews, R.M. & Bramble, J.E. 1992. Size and growth rate characteristics of a peripheral population of *Crotaphytus collaris* (Sauria: Crotaphytidae). *Copeia* **1992**:968-980.

Shine, R. 1986. Sexual differences in morphology and niche utilization in an aquatic snake, *Acrochordus arafurae*. *Oecologia***69**:260-267.

Shine, R. 1977a. Reproduction in Australian elapid snakes I. Testicular cycles and mating seasons. *Aust. J. Zool.***25**:647-653.

Shine, R. 1977b. Reproduction in Australian elapid snakes II. Female reproductive cycles. Aust. *J. Zool.***25**:655-666.

Shine, R. 1978. Growth rates and sexual maturation in six species of Australian elapid snakes. *Herpetologica* **34**:73-79.

Sivan, J., Kam, M., Hadad, S., Degen, A.A., Rozenboim, I. & Rosenstrauch, A. 2012. Reproductive cycle of free-living male Saharan sand vipers, *Cerastes vipera* (Viperidae) in the Negev desert, Israel. *Gen. Comp. Endocr.* **179**:241-247.

Solórzano, A. & Cerdas, L. 1989. Reproductive biology and distribution of the terciopelo, *Bothrops asper* Garman (Serpentes: Viperidae), in Costa Rica. *Herpetologica* **45**:444-450.

Stamps, J.A., Losos, J.B. & Andrews, R.M. 1997. A comparative study of population density and sexual size dimorphism in lizards. *Am. Nat.* **149**:64-90.

Steen, D.A., Smith, L.L., Conner, L., Brock, J.C. & Hoss, S.K. 2007. Habitat use of sympatric rattlesnake species within the Gulf Coastal Plain. *J. Wildlife Manage.* **71**:759-764.

Tang, X.S., Lu, S.Q. & Chou, W.H. 2007. Description of male *Takydromus sylvaticus* (Squamata: Lacertidae) from China, with notes on sexual dimorphism and a revision of the morphological diagnosis of the species. *Zool. Sci.***24**:496-503.

Tollestrup, K. 1982. Growth and reproduction in two closely related species of leopard lizards, *Gambelia silus* and *Gambelia wislizenii*. *Am. Midl. Nat*. **108**:1-20.

Trauth, S.E. 1979. Testicular cycle and timing of reproduction in the collared lizard (*Crotaphytus collaris*) in Arkansas. *Herpetologica* **35**:184-192.

Valdéz-González, M.A. & Ramírez-Bautista, A. 2002. Reproductive characteristics of the spiny lizards, *Sceloporus horridus* and *Sceloporus spinosus* (Squamata: Phrynosomatidae) from México. *J. Herpetol.* **36**:36-43.

Van Sluys, M. 1993a. Food habits of the lizard *Tropidurus itambere* (Tropiduridae) in southeastern Brazil. *J. Herpetol.* **27**:347-351.

Van Sluys, M. 1993b. The reproductive cycle of *Tropidurus itambere* (Sauria: Tropiduridae) in southeastern Brazil. *J. Herpetol.* **27**: 28-32.

Van Sluys, M. 1998. Growth and body condition of the saxicolous lizard *Tropidurus itambere* in southeastern Brazil. *J. Herpetol.* **32**:359-365.

Van Sluys, M. 2000. Population dynamics of the saxicolous lizard *Tropidurus itambere* (Tropiduridae) in a seasonal habitat of southeastern Brazil. *Herpetologica* **56**:55-62.

Vitt, L.J. 1991. Ecology and life history of the wide-foraging lizard *Kentropyx calcarata* (Teiidae) in Amazonian Brazil. *Can. J. Zoolog.* **69**:2791-2799.

Vitt, L.J. 1993. Ecology of isolated open-formation *Tropidurus* (Reptilia: Tropiduridae) in Amazonian lowland rain forest. *Can. J. Zoolog.* **71**:2370-2390.

Vitt, L.J. & Blackburn, D.G. 1991. Ecology and life history of the viviparous lizard *Mabuya bistriata* (Scincidae) in the Brazilian Amazon. *Copeia* **1991**:916-927.

Vitt, L.J. & de Carvalho, C.M. 1995. Niche partitioning in a tropical wet season: lizards in the lavrado area of northern Brazil. *Copeia* **1995**:305-329.

Vitt, L.J. & Cooper Jr., W.E. 1985a. The evolution of sexual dimorphism in the skink *Eumeces laticeps*: an example of sexual selection. *Can. J. Zoolog.* **63**:995-1002.

Vitt, L.J. & Cooper Jr., W.E. 1985b. The relationship between reproduction and lipid cycling in the skink *Eumeces laticeps* with comments on brooding ecology. *Herpetologica* **41**:419-432.

Vitt, L.J. & Goldberg, S.R. 1983. Reproductive ecology of two tropical iguanid lizards: *Tropidurus torquatus* and *Platynotus semitaeniatus*. *Copeia* **1983**:131-141.

Vitt, L.J. & Ohmart, R.D. 1977. Ecology and reproduction of Lower Colorado River lizards: II*. Cnemidophorus tigris* (Teiidae), with comparisons. *Herpetologica* **33**:223-234.

Vitt, L.J., Sels, R.C.V.L. & Ohmart., R.D. 1978. Lizard reproduction: annual variation and environmental correlates in the iguanid lizard *Urosaurus graciosus*. *Herpetologica* **34**:241-253.

Vitt, L.J., Zani, P.A., Caldwell, J.P., De Araujo, M.C. & Magnusson, W.E. 1997. Ecology of whiptail lizards (*Cnemidophorus*) in the Amazon region of Brazil. *Copeia* **1997**:745-757.

Voris, H.K. & Jayne, B.C. 1979. Growth, reproduction and population structure of a marine snake, *Enhydrina schistosa* (Hydrophiidae). *Copeia* **1979**:307-318.

White, M. & Kolb, J.A. 1974. A preliminary study of *Thamnophis* near Sagehen Creek, California. *Copeia* **1974**:126-136.

White, D.R., Mitchell, J.C. & Woolcott, W.S. 1982. Reproductive cycle and embryonic development of *Nerodia taxispilota* (Serpentes: Colubridae) at the northeastern edge of its range. *Copeia* **1982**:646-652.

Wilhoft, D.C. & Quay, W.B. 1961. Testicular histology and seasonal changes in the lizard, *Sceloporus occidentalis*. *J. Morphol.* **108**:95-106.

Whitaker, P.B. & Shine, R. 2003. A radiotelemetric study of movements and shelter-site selection by free-ranging brownsnakes (*Pseudonaja textilis*, Elapidae). *Herpetol. Monogr*. **17**:130-144.
